# Characterization of two putative potassium channels in *Plasmodium falciparum*

**DOI:** 10.1186/1475-2875-7-19

**Published:** 2008-01-24

**Authors:** Karena L Waller, Sean M McBride, Kami Kim, Thomas V McDonald

**Affiliations:** 1 Department of Medicine (Cardiology), Albert Einstein College of Medicine, 1300 Morris Park Ave., Bronx NY 10461, USA; 2 Department of Molecular Pharmacology, Albert Einstein College of Medicine, Bronx NY 10461, USA; 3 Department of Medicine (Infectious Diseases), Albert Einstein College of Medicine, Bronx NY 10461, USA; 4 Department of Microbiology and Immunology, Albert Einstein College of Medicine, Bronx NY 10461, USA; 5 Department of Microbiology, Monash University, Clayton VIC 3800, Australia

## Abstract

**Background:**

Potassium channels are essential for cell survival and participate in the regulation of cell membrane potential and electrochemical gradients. During its lifecycle, *Plasmodium falciparum *parasites must successfully traverse widely diverse environmental milieus, in which K^+ ^channel function is likely to be critical. Dramatically differing conditions will be presented to the parasite in the mosquito mid-gut, red blood cell (RBC) cytosol and the human circulatory system.

**Methods:**

*In silico *sequence analyses identified two open-reading frames in the *P. falciparum *genome that are predicted to encode for proteins with high homology to K^+ ^channels. To further analyse these putative channels, specific antisera were generated and used in immunoblot and immunofluorescence analyses of *P. falciparum*-infected RBCs. Recombinant genome methods in cultured *P. falciparum *were used to create genetic knock outs of each K^+ ^channel gene to asses the importance of their expression.

**Results:**

Immunoblot and IFA analyses confirmed the expression of the two putative *P. falciparum *K^+ ^channels (PfK1 and PfK2). PfK1 is expressed in all asexual stage parasites, predominantly in late stages and localizes to the RBC membrane. Conversely, PfK2 is predominantly expressed in late schizont and merozoite stage parasites and remains primarily localized to the parasite. Repeated attempts to knockout PfK1 and PfK2 expression by targeted gene disruption proved unsuccessful despite evidence of recombinant gene integration, indicating that *pfk1 *and *pfk2 *are apparently refractory to genetic disruption.

**Conclusion:**

Putative K^+ ^channel proteins PfK1 and PfK2 are expressed in cultured *P. falciparum *parasites with differing spatial and temporal patterns. Eventual functional characterization of these channels may reveal future pharmacological targets.

## Background

In the course of a persistent human infection, *Plasmodium falciparum *parasites are exposed to dramatic changes in the extracellular milieu in terms of pH, osmolarity and ionic constituents. To survive such conditions transporter pumps, exchangers and ion channels operate to maintain the intra-parasite environment in the face of varying external conditions. Many classes of putative *P. falciparum *channels and transporters have been identified [1]. These are thought to be critical for parasite viability [2,3] and offer potential new molecular targets for the development of novel anti-malarials.

**Figure 1:**
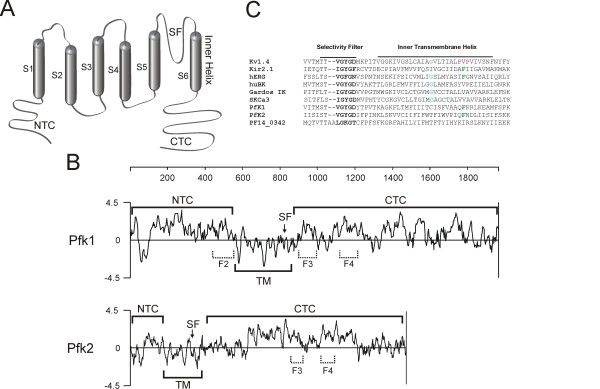
**PfK1 and PfK2**. **A**. Membrane topology schematic of a single K^+ ^channel subunit. Predicted topographical domains are indicated: NTC = N-terminal cytoplasmic portion; CTC = C-terminal cytoplasmic portion; SF = selectivity filter; and S1–S6 = membrane spanning domains. **B**. Hydropathy plots of the predicted protein sequences of PfK1 and PfK2. Negative deflections signify hydrophobic sequences. NTC = N-terminal cytoplasmic portion; CTC = C-terminal cytoplasmic portion; TM = Transmembrane portion and SF = selectivity filter. F2, F3 and F4 refer to protein segments used to generate specific antisera. **C**. Alignment of channel conduction pore protein sequences of various well characterized human K^+ ^channels, highlighting features common to the family (**G **= suggested flexibility point for voltage-gated K^+ ^channels. **P **= possible bend-inducing prolines. **F **= sites for possible drug block). Kv1.4 = voltage-gated Shaker-type K^+ ^channel, Kir2.1 = inward rectifying K^+ ^channel, hERG = human Ether a-gogo-Related Gene channel, huBK = human BK Ca^2+^-activated K^+ ^channel, Gardos IK K^+ ^channel and SKCa3 SK Ca^2+^-activated K^+ ^channel. The sequence encoded by PF14_0342 is also included in the alignment, due to its suggestion as being a possible third parasite-encoded K^+ ^channel [1]. PF14_0342 does not have several features common to K^+ ^channels, including the pore region GYG motif.

One class of transporters, the potassium (K^+^) channels, are transmembrane proteins that gate open and closed to control the flow of K^+ ^ions across cell membranes. K^+ ^channels are critical in the regulation of the transmembrane electrochemical gradient, cell membrane potential and intracellular osmolarity and are essential for the survival of all known cells. K^+ ^channels share several common features. They have 2, 4 or 6 membrane-spanning regions, two of which contribute to form the "pore region" through which K^+ ^ions move (Figure 1A). A highly conserved GYG (or the less common GFG) signature motif within the pore region comprises the ion selectivity filter [4]. Functional channels result from a tetrameric complex of identical or closely related subunits, with the ion conduction pathway formed by the four-fold symmetry of the component subunits.

Here, a bioinformatic approach was used to identify two putative K^+ ^channel genes (*pfk1 *and *pfk2*). This report details the cellular investigation of these putative K^+ ^channel proteins, showing their differential expression and cellular localization in asexual blood stage parasites. The identification of only two putative K^+ ^channels encoded within the *P. falciparum *genome, in conjunction with their differing expression and localization profiles suggests that these proteins may play unique and potentially critical roles in the maintenance of parasite viability.

## Methods

### Parasite culture

*Plasmodium falciparum *3D7 parasites were maintained by *in vitro *culture using standard culture conditions [5] and supplemented with 0.5% Albumax II (Invitrogen). Parasitaemias were determined by microscopic examination of Giemsa stained thin blood smears.

### Homology searches, molecular coning and knockout parasite transfection

Homology searches of the *P. falciparum *3D7 genome database [6] were performed using the conserved K^+ ^channel GYG selectivity filter motif. Extracted sequences were further scrutinized to identify any predicted flanking membrane-spanning segments. Two gene sequences, designated *pfk1 *(PFL1315w) and *pfk2 *(PF14_0622), were identified and amplified from genomic *P. falciparum *3D7 DNA by PCR using specific primers (Table 1). All nucleotide sequences were confirmed by automated sequence analysis (Genewiz Inc.).

**Table 1:** Oligonucleotide primers.

**Primer**	**Sequence (5'→ 3')**^*a*^	**Target**^*b*^
P1	cgggaattcATGAATAATGATAATATTGGGAG	*pfk1 *full length (+)
P2	cccaagcttTCAGACTTGGTCATGCGGTTC	*pfk1 *full length (-)
P3	cgggaattcGTTGATAAGAAAGGAAAAATAATAGAT	*pfk1 *F2 (+)
P4	cccaagcttATGTACAAATCGATGTTTTGTATA	*pfk1 *F2 (-)
P5	ccgggaattcatTTTAATAATCATGATCATCAGAATC	*pfk1 *F3 (+)
P6	cccaagcttTAAACATTCTTGATCATCTTTTTCC	*pfk1 *F3 (-)
P7	ccgggaattctcAAAAAAGGACAAAACCATCAACTT	*pfk1 *F4 (+)
P8	cccaagcttCATATTTTTTTCCATATCTTTAATG	*pfk1 *F4 (-)
P9	cgcggatccgATGAAAAGCGGATTATTTTCTATG	*pfk2 *full length (+)
P10	ccggaattcTCACAATATATAAACTATATCATCGAATC	*pfk2 *full length (-)
P11	ccgggaattcATAATGATAATGATAATAATAATATTATA	*pfk2 *F3 (+)
P12	cccaagctttTTTAATTCCTTTTTATTATCAATTGT	*pfk2 *F3 (-)
P13	ccgggaattcGAATATCAAGAATATTTACCAAAAAC	*pfk2 *F4 (+)
P14	cccaagcttATTATGAATATGAATATTTGTATTTGA	*pfk2 *F4 (-)
P15	aaactgcagTGAAAACAGAATGCGATTAGTTCG	*pfk1 *KO (+)
P16	cgcggatccGATGATGCAAATGTTAATGGTAC	*pfk1 *KO (-)
P17	aaactgcagTTCTTATCTTATTGAACGGATCTC	*pfk2 *KO (+)
P18	cgcggatccTTGTATGGTATATATAGTAGATGC	*pfk2 *KO (-)
P19	GATAGCGATTTTTTTTACTGTCTG	*hrp2 *3'UTR (+)
P20	GAAATTAACCCTCACTAAAGGG	plasmid backbone
P21	aaggaaaaaagcggccgcAATGAATAATGATAATATTGGGAG	*pfk1 *(+)
P22	cccaagcttAGATATAATACCATCCGTTTGG	*pfk1 *(-)
P23	ccggaattcAATGAAAAGCGGATTATTTTCTATG	*pfk2 *(+)
P24	cgcggatccTTGTATGGTATATATAGTAGATGC	*pfk2 *(-)
P25	TTGTTGTGGTTCTACATCTCC	*pfk2 *(-)
P26	CACGAAGCCGCCACACATTGCC	*hrp2 *orf1
P27	TTTGATCTTGTTCACTATGGC	*pfk1 *(-)
P28	cccaagcttATTACAAAAATAATAATTGAAATTACT	*pfk2 *(-)
P29	GTAATACGACTCACTATAGGGC	plasmid backbone
P30	TTGCAATTCTGCTTCAGTTGG	*cam *(-)

^*a *^Sequences shown in upper case are gene specific. Underlined sequences indicate enzyme restriction sites.

^*b *^+ and - indicate sense and anti-sense strand sequences, respectively.

Approximately 1.0 kb regions of *pfk1 *and *pfk2 *(downstream of, but not including the endogenous ATG start site) were amplified by PCR (Table 1) and cloned into the transfection plasmid pminiBSD [7], generating the plasmids pminiBSD/*pfk1*-KO and pminiBSD/*pfk2*-KO. Transfection of *P. falciparum *3D7 parasites was performed using previously described methods [8,9]. Transfected cultures were screened by PCR for plasmid integration [10] using specific primers (Table 1) approximately three months after transfection. Integration positive cultures were then cloned by limiting dilution [10].

### Western blotting and indirect immunofluorescence assays (IFA)

Recombinant Glutathione *S*-transferase (GST) fusion proteins of PfK1 and PfK2 were generated. Regions of *pfk1 *and *pfk2 *(Figure 1B) were cloned into pGEX-KG and the encoded fusion proteins expressed and purified using standard affinity chromatography [11]. Rabbit polyclonal antibodies were generated (Covance), and affinity purification was performed using the GST-fusion proteins immobilized on CN-Br Sepharose (Amersham Biosciences). The paired pre-immune control sera were obtained prior to immunization of the rabbits, and were used in simultaneous immunochemical methodologies (see above) as controls. No cross-reactivity was observed.

Parasite lysates were prepared from cultures synchronized by sorbitol lysis [12]. Cultures were sampled at regular intervals and the cells lysed using 0.15% saponin in phosphate-buffered saline (PBS; 137 mM NaCl, 2.7 mM KCl, 4.3 mM Na_2_HPO_4_, 1.4 mM KH_2_PO_4_, pH7.4). Parasites were washed in STE (100 mM NaCl, 20 mM Tris-HCl pH8.0, 50 mM EDTA), before resuspending in reducing SDS-PAGE loading buffer and incubation at 37°C for 30 minutes. Samples were not boiled to facilitate solubility and prevent aggregation of transmembrane proteins [13,14]. Proteins were resolved in 7.5% or 4–15% gradient polyacrylamide gels (Bio-Rad Laboratories) before transfer to nitrocellulose membranes. Western blotting was performed using affinity purified anti-PfK1 or anti-PfK2 antibodies (1/500) and detected using Western Lightning Chemiluminescence Reagent Plus (PerkinElmer Life Sciences). Control western blots were performed using polyclonal anti-KAHRP (provided by R. L. Coppel) and anti-GRP(BiP) antisera ([15]; MRA20, MR4 ATCC).

Cellular localization of PfK1 and PfK2 by IFAs was performed on sorbitol-synchronized parasites. Thin blood smears were prepared from cultures at approximately 12-hour intervals before fixation with methanol/acetone (9:1). Slides were incubated with affinity purified antibodies in PBS containing 0.5% Bovine Serum Albumin (BSA) for 30 minutes, washed extensively with PBS and stained with goat anti-rabbit AlexaFluor^®^488 conjugate antibody (1/1000; Molecular Probes). For double-labelled IFAs, thin blood smears were prepared in the same manner and sequentially incubated with primary antibodies before staining with goat anti-rabbit AlexaFluor^®^488 and goat anti-mouse AlexaFluor^®^594 conjugate antibodies (both 1/1000; Molecular Probes). Parasite nuclei were visualized with 4',6-Diamidino-2-phenylinodole dihydrochloride (DAPI; 10 μg/ml). Slides were washed and mounted in Fluoromount-G (Southern Biotech). Cells were visualized using an Olympus IX81 electronically motorized microscope. Images were captured using a Sensicam QE cooled CCD camera (The Cooke Corporation) and IP Lab Spectrum Scientific Imaging Processing Software (Scanalytics Inc.). Images were then deconvolved using Vaytek Image software (Vaytek Inc.). The primary antibodies were: affinity purified anti-PfK1 F4 (1/250) and anti-PfK2 F4 (1/100), polyclonal rabbit anti-KAHRP (1/250) and anti-MESA (1/250 [11] ; provided by R. L. Coppel) and mouse mAb5.2 anti-MSP1 (1/100; MRA94, MR4 ATCC).

## Results

### Identification of *pfk1 *and *pfk2*

Searches of the *P. falciparum *3D7 genome database using the conserved K^+ ^channel selectivity filter GYG motif yielded two gene sequences, designated here *pfk1 *(PFL1315w, or *pfkch1 *[16]) and *pfk2 *(PF14_0622). *Pfk1 *is approximately 6.1 kb and is located on chromosome 12. The *pfk2 *gene is approximately 4.4 kb and located on chromosome 14. Both genes possess a single exon and are predicted to encode highly charged proteins; PfK1 is rich in asparagine, lysine, arginine, glutamic and aspartic acid, whereas PfK2 is rich in asparagine, lysine, leucine, isoleucine and tyrosine. Both protein sequences are predicted to have six membrane-spanning regions and a pore region (Fig. 1B). Alignments of the pore region sequence from PfK1 and PfK2 with other well characterized K^+ ^channels demonstrates overall similarity between these K^+ ^channel proteins (Figure 1C), with closest similarity observed within the pore region of Ca^2+^-activated K^+^channels.

### Differential expression of PfK1 and PfK2

**Figure 2:**
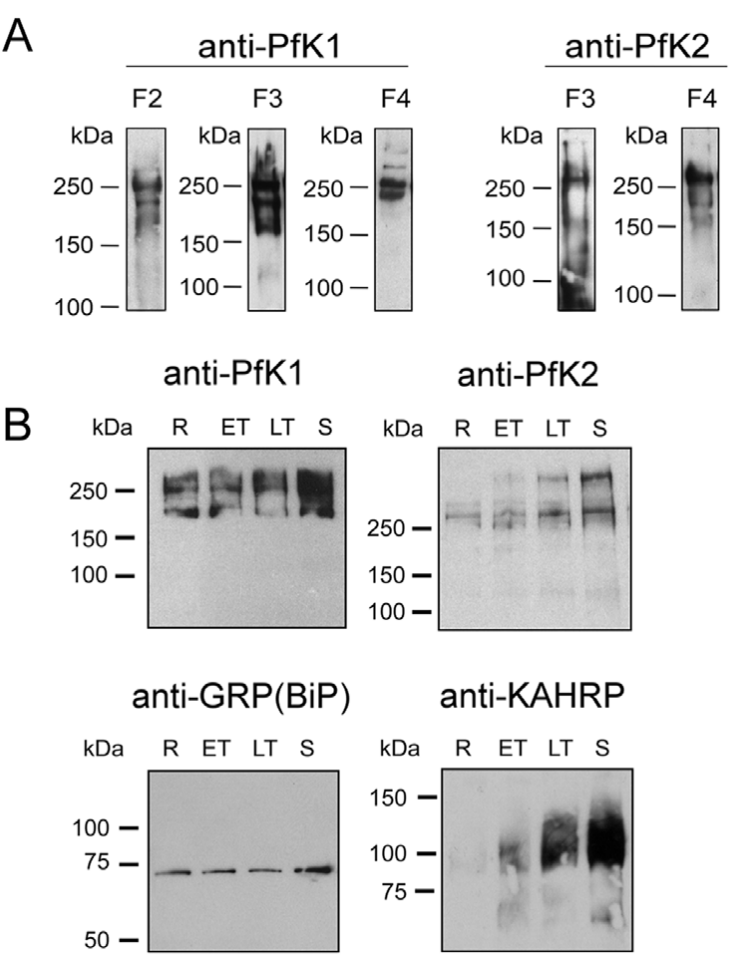
**Expression of PfK1 and PfK2**. **A**. Immunoblots of identical synchronized parasite samples (loading 2 × 10^7 ^parasites per lane; rich in schizonts and early rings) probed with each of the three anti-PfK1 and two anti-PfK2 antibodies. Similar banding profiles are detected by each anti-PfK1 or anti-PfK2 antibodies. **B**. Synchronous cultures were sampled over the 48 hr lifecycle and probed by Western Blot (loading ~1 × 10^7 ^parasites per lane). PfK1 was detected as a triplet set of bands of >200 kDa using anti-PfK1 F3 antibodies. Expression of PfK1 was detected across the 48 hr lifecycle. PfK2 was detected as two bands of >250 kDa using anti-PfK2 F4 antibodies, with maximal expression detected in late schizonts (ie. samples rich in developing merozoites). Constitutive expression of the ER marker protein GRP(BiP) across the 48 hr lifecycle of *P. falciparum *was detected at approximately 70 kDa. Stage specific expression of KAHRP, observed as a broad band of reactivity at ~100 kDa, with maximal expression observed in mature trophozoites and schizonts. Minimal expression was detected in samples rich in ring stage parasites. The samples are: rings (R), early trophozoites (ET), late trophozoites (LT) and schizonts (S).

Immunoblots of synchronized parasites were performed using affinity purified antibodies specific for PfK1 and PfK2 (Figure 2A) and pre-immune control sera (no cross-reactivity was observed). Initially, three different anti-PfK1 and two anti-PfK2 antibodies were used to probe identical synchronized parasite samples. In each case, PfK1 expression was detected as a group of three high molecular mass bands of >200 kDa, whereas PfK2 expression was detected as a doublet of >250 kDa band (Figure 2A). Further, immunoblots of time-course parasite material showed PfK1 was expressed throughout the 48 hr asexual lifecycle, but greater expression levels were detected in more mature parasites. PfK2 abundance also varied through the 48 hr time course, with maximal expression being detected in samples rich in merozoites and late schizonts (Figure 2B). The multiple bands of PfK1 and PfK2 may be attributed to incomplete disruption of PfK oligomers into individual subunits or from association of other K^+ ^channel regulatory subunits during the relatively mild sample preparation conditions.

Additional blots were performed in parallel using antibodies against the endoplasmic reticulum (ER) marker protein Glucose-regulated protein GRP(BiP) [15] and the Knob Associated Histidine Rich Protein (KAHRP; Fig. 2). GRP(BiP) mRNA has been shown by microarray to be constitutively expressed across the 48 hr asexual life cycle [17-19]. Conversely, KAHRP is expressed by intracellular parasites in a stage-specific manner (see Cooke *et al *for review [20]). GRP(BiP) was readily detected in all samples at approximately equal levels, whereas maximal KAHRP expression was detected in mature trophozoites and schizonts.

### Differential localization of PfK1 and PfK2

**Figure 3:**
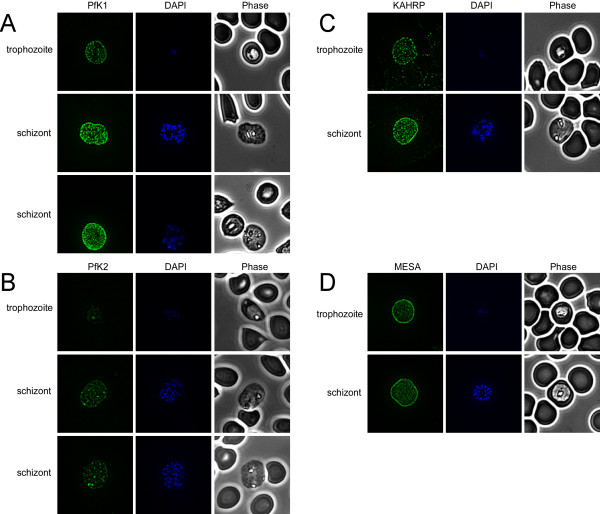
**Localization of PfK1 and PfK2**. Indirect Immunofluorescence Assays (IFAs) were performed using affinity purified anti-PfK1 F4 and -PfK2 F4 antibodies, and anti-KAHRP and anti-MESA antisera. Representative IFAs are shown for each antibody. **A **Anti.-PfK1 IFAs showed no labeling in rings (data not shown) or uRBCs, and increasing RBC membrane labeling in trophozoites and schizonts. Predominantly labeling occurs in a semi-punctate localization pattern. **B**. PfK2 was not detected in rings (data not shown) or uRBCs, and minimal levels were detected in trophozoites. Schizonts showed intense foci of labeling, which appeared to correspond with the location of the developing merozoites within RBCs. **C**. KAHRP was detected in a classic punctuate pattern on the surface of trophozoites and schizonts. No labeling of KAHRP was detected in ring-iRBCs (data not shown). **D**. More diffuse RBC membrane labeling of MESA was detected on trophozoites and schizonts. No labeling was detected in rings (data not shown).

Although detectable by Western blotting, PfK1 was not detected by IFA in ring infected red blood cells (iRBCs) or in surrounding uninfected red blood cells (uRBCs; Figure 3). However, IFA labeling was detected in trophozoites and schizonts (using anti-PfK1 F4 antibodies). The level of labeling increased with parasite maturity and was located at the plasma membrane of the iRBC, in a semi-punctate pattern (Figure 3A). Intense punctate labeling of PfK2 was detected in mature schizonts using anti-PfK2 F4 antibodies, but not in uRBCs (Figure 3B), in a pattern suggesting co-localization with the developing merozoites within the schizont. Anti-PfK2 F4 antibodies failed to label rings, with minimal labeling detected in trophozoites. Similar IFA results for both PfK1 and PfK2 localization were obtained using the alternate antibodies anti-PfK1 F3 and anti-PfK2 F3, respectively, and pre-immune control sera showed not cross-reactivity.

Control IFAs were performed with KAHRP and Mature parasite-infected Erythrocyte Surface Antigen (MESA) antisera. KAHRP is exported into the red blood cell (RBC) cytosol where it associates with the membrane skeleton, forming electron-dense, knob-like structures at the RBC membrane. MESA is exported from the intracellular parasite into the RBC cytosol where it associates with the membrane skeleton, but has no specific association with knobs (see Cooke *et al *for review [20]). Increasing intensity of punctate KAHRP labeling was observed with increasing parasite maturity, with maximal labeling detected on the RBC membrane of trophozoites and schizonts (Figure 3C). Increased MESA labeling was observed at the RBC membrane of the mature trophozoites and schizonts with a nearly confluent appearance (Figure 3D). The pattern of PfK1 labeling more closely resembled the punctate appearance of the RBC membrane-associated protein KAHRP, rather than that of MESA.

**Figure 4:**
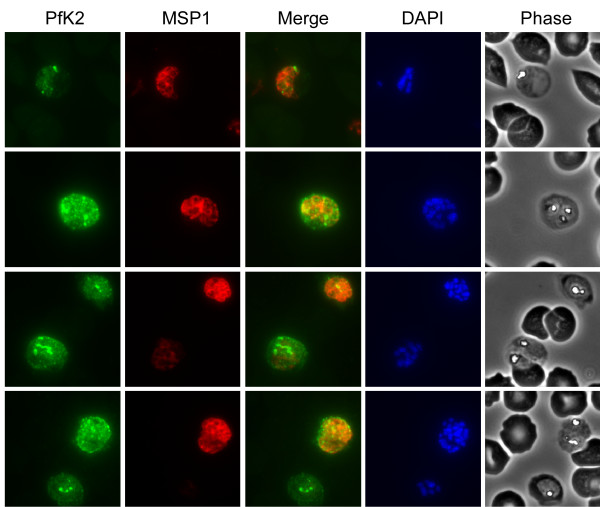
**Localization of PfK2 in schizont-infected RBCs**. Indirect Immunofluorescence Assays (IFAs) were performed using affinity purified rabbit anti-PfK2 F4 antibodies (green), and mouse monoclonal anti-MSP1 (red). Individual and merged PfK2 and MSP1 IFA panels are shown, in addition to the corresponding DAPI-labeled and phase images. Schizonts showed intense foci of labeling for PfK2, which appeared to correspond with the location of the developing merozoites within RBCs. In some cases, less intense PfK2 labeling was observed outside of the merozoites and in the RBC cytosol.

To further define the localization of PfK2, double-labeled IFAs using antibodies against the merozoite markers MSP1 (Figure 4) and MSP4 were performed. The most intense staining suggests that PfK2 is associated directly with the parasite. In schizonts, labeling was primarily confined to the parasite itself, with small intensely labeled regions appearing to co-localize with developing merozoites. Lower levels of diffuse labeling of PfK2 in inter-merozoite regions and RBC cytoplasm were also detected in some iRBCs. However, resolution by IFA was not sufficient to definitively determine whether there was RBC membrane association. Numerous localization attempts using immuno-electron microscopy failed with affinity purified antibodies specific for both PfK1 and PfK2.

### Pfk1 and pfk2 knockout (KO) transfections

**Figure 5:**
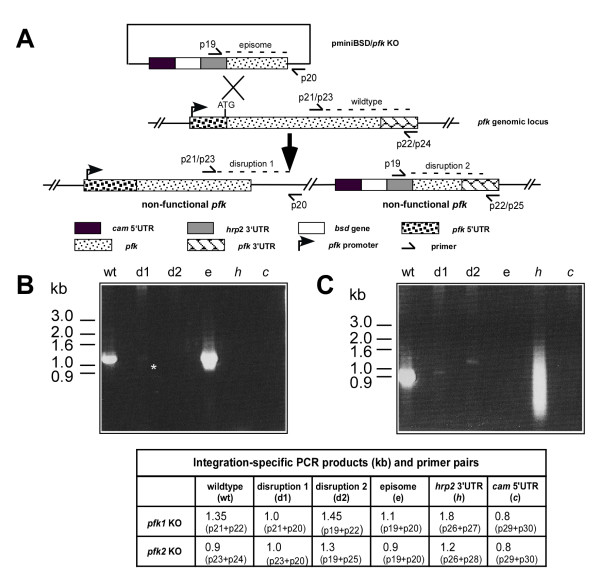
***pfk1 *and *pfk2 *knockout transfections**. **A**. Schematic representation of the knockout transfection plasmid (pminiBSD/*pfk *KO), *pfk *loci within the parasite genome, and the resultant *pfk *loci after successful integration. Relative locations of primers used in the PCR detection of integration are shown. Integration-specific PCR products of *pfk1 *(**B**.) and *pfk2 *(**C**.) are shown. Expected sizes of PCR products resulting from each primer pair are shown in the table below. A ~1.0 kb product (*) was detected in d1, indicative of integration into *pfk1 *(panel B), whereas 1.0 kb (d1) and 1.3 kb (d2) products indicative of integration into *pfk2 *(panel C) were detected. Integration into the genes *hrp2 *and *cam *was also assessed, as regulatory elements of these *Plasmodium sp. *genes drive expression of the *bsd *gene present in the drug selection cassette on the plasmid. Integration into the *hrp2 *loci was detected in the *pfk2 *transfection culture only (panel C, lane *h*).

Genetic disruption or knockout (KO) of *pfk1 *and *pfk2 *expression was attempted to examine the importance of these K^+ ^channels in parasite viability *in vitro*. After transfection, PCRs were performed to demonstrate the presence of KO disrupted *pfk1 *and *pfk2 *parasites in transfection cultures (Figure 5). In each case, PCR amplification of the expected size DNA products demonstrated integration into both the *pfk1 *or *pfk2 *loci in bulk transfection cultures (Figure 5, panels B and C, lanes d1 and d2), but despite repeated attempts, no clones were isolated after cloning by limited dilution. That *pfk1 *and *pfk2 *KO integration can be detected via PCR in bulk transfection cultures, but a parasite clone could not be isolated suggests that disruption of these genes is deleterious to parasite viability.

## Discussion

Asexual *P. falciparum *parasites must rapidly respond to the widely variant ionic conditions, pH and osmolarities of their external milieus. In order to adapt to these environments, parasites most likely utilize ion channels and transporters to facilitate their regulation of intracellular ion concentrations. Allen and Kirk have shown that the transmembrane potential across the *Plasmodium *parasite plasma membrane is primarily generated by an electrogenic proton pump [21]. The proton efflux in this study was balanced by influx of K^+^, presumably through such surface channels. This K^+ ^transport may be carried by PfK1 and/or PfK2. During blood-stage development, various parasite proteins are synthesized and exported into the RBC resulting in modification of normal RBC structure and function [20]. New permeation pathways (NPP) are hypothesized to facilitate the transport of low molecular mass solutes [2,3] from the RBC membrane to the parasite and are likely to include many of the predicted parasite-encoded channels and transporters encoded in the parasite genome [1].

K^+ ^channels are essential for the viability of all cells and are likely to be critical for malaria viability. The *P. falciparum *3D7 genome encodes only two putative K^+ ^channel genes, *pfk1 *and *pfk2*. The search criteria employed in this study specified that the selectivity filter motif be flanked by at least two predicted membrane-spanning segments. Although the number of transmembrane domains can vary with K^+ ^channel type, in all cases, a single ion channel is created by the assembly of a combination of subunits that contribute four inner helices and four selectivity filter domains. Voltage-gated and Ca^2+^-activated K^+ ^channels contain six (or seven, in the case of big conductance (BK) K^+ ^channel) membrane spanning segments with the ion selectivity filter between the 5^th ^and 6^th ^membrane segment. Both PfK1 and PfK2 are predicted to have six membrane-spanning domains, and a pore region between the 5^th ^and 6^th ^segments based on hydropathy analysis and sequence alignments (Figure 1). The predicted pore-forming structures of PfK1 and PfK2 most resemble those of Ca^2+^-activated K^+ ^channels. PfK1 also has a cluster of evenly spaced arginines in the 4^th ^membrane-spanning segment that suggests the possibility of voltage-sensitivity. PfK2 lacks these charged residues in the 4^th ^transmembrane segment and is thus more akin to intermediate conductance (IK) or small conductance (SK) channels. Full protein sequence alignments of PfK1 and PfK2 with other characterized K^+ ^channels show less sequence similarity outside of the conserved pore-forming regions (data not shown), however their overall topologies are consistent with other voltage-gated and Ca^2+^-activated K^+ ^channels.

A possible third *P. falciparum *K^+ ^channel gene, PF14_0342, has been suggested from searches detailed by Martin *et al *[1]. This sequence is unlikely to encode a K^+ ^channel due its lack of several key features that are common to many K^+ ^channels. The predicted selectivity filter contains a GKG motif instead of GYG (Fig. 1C) and lacks appropriate flanking transmembrane regions.

Here, the differential expression and localization of two putative *P. falciparum *potassium channels have been demonstrated. Recent global gene expression studies show *pfk1 *mRNA detectable throughout the entire 48-hour asexual cycle [17-19,22]. In support of these data, PfK1 was detected by immunoblot in all RBC stages, though more protein was detected in mature forms. By IFA, PfK1 expression was detected from early trophozoites through schizont stage parasites. In ring stage parasites, the density of PfK1 may be too low, such that its detection was beyond the sensitivity of IFA. More intense labeling in mature-iRBCs (Figure 3) and also in multiply-iRBCs was consistently observed.

Like KARHP and MESA, PfK1 localizes to the RBC membrane. It is hypothesized that PfK1 is expressed by the parasite and exported into the RBC, where it is inserted into the RBC plasma membrane. Recent bioinformatic analyses of trafficked protein sequences have identified a conserved PEXEL/VTS motif that targets proteins for trafficking into the RBC [23,24], in addition to the SS motif that directs proteins via the ER into the secretory pathway. *In silico *analysis [24,25] of the PfK1 protein sequence for motifs indicated as potential export/transport signals failed to identify a VTS-like sequence, however manual searches yielded two potential VTS sequences, FFYRKLKNTFM and NFRRFLSSYKS (located 99 and 169 residues from the N-terminus, respectively). PfK1 also contains a predicted SS sequence (YDKRFSSRIKPK), located upstream of the VTS motifs and 20 residues from the N terminus of PfK1 (K. Haldar, personal communication).

PfK2 is primarily expressed in late schizonts and merozoites, in agreement with previous gene expression studies [18,19,23]. By IFA, PfK2 was detected in some, but not all, young ring stage parasites. Manual analysis of the PfK2 sequence revealed the presence of a possible SS cleavage motif and a low value VTS motif, but their configuration and location within the protein make it unclear whether these could function in the export of PfK2 (K. Haldar, personal communication).

From its localization, it is hypothesized that PfK1 encodes a parasite-induced ion channel that results in modification of the normal RBC to facilitate intracellular survival and maturation of the parasite by virtue of regulating K^+ ^flux across the iRBC membrane, thereby effecting maintenance of the membrane potential and electrochemical gradient across the RBC plasma membrane and composition of the RBC cytosol.

PfK2, which is expressed predominantly in the merozoite, may function in the maintenance of membrane potential and the electrochemical gradient across merozoite membranes that are exposed to the human circulatory system. It is possible that PfK2 may act in concert with PfK1 during this time to adapt to environment changes the parasites are exposed to when released from the rupturing RBC. Invasion studies using the Apicomplexan parasite *Toxoplasma gondii *have shown that host cell invasion is accompanied by fluctuations of intracellular Ca^2+ ^[26,27], similar to those described during antigen-stimulated activation and proliferation of T cells. In those cells, effective Ca^2+ ^signaling and ultimately downstream gene expression are modified by K^+ ^channel activity to maintain the cell membrane potential [28]. By analogy, it is hypothesized that PfK2 is a merozoite-specific K^+^channel that functions during the process of merozoite invasion of RBCs, perhaps functioning in ways to similarly effect intracellular Ca^2+ ^signaling and gene transcription in the invading merozoite.

Heterologous expression and patch-clamp studies are required to definitively assign PfK1 and PfK2 to a class of K^+ ^channels. Functional expression of both native and codon-optimized PfK1 and PfK2 cDNAs in a variety of heterologous systems has so far proven unsuccessful (McBride and McDonald, unpublished data). Expression of active parasite-encoded K^+ ^channels in heterologous systems would illuminate the potential function of PfK1 and PfK2. Such a system could be adapted for the screening of novel specific blockers in a high through-put format for identification of possible new anti-malarials.

To examine the importance of these K^+ ^channels for parasite survival, attempts were made to knockout *pfk1 *and *pfk2 *expression. The ability to detect 'snap shots' of integration by PCR and inability to isolate parasite clones despite repeated attempts are consistent with the hypothesis that both *pfk1 *and *pfk2 *are critical for parasite viability. Failed attempts to disrupt various parasite genes thought to be necessary for parasite invasion, viability or propagation have been reported [10,29-31]. Further experimentation using either specific gene expression knockdown [10] or tetracycline-dependant conditional expression systems [32] may help unravel the precise functional roles of PfK1 and PfK2 in the regulation of K^+ ^physiology in malaria-iRBCs.

## Abbreviations

K^+^, Potassium Ion; RBC, Red Blood Cell; IFA, Indirect Immunofluorescence Assay; PfK, *Plasmodium falciparum *Potassium channel; PCR, polymerase chain reaction; GST, Glutathione *S*-transferase; PBS, Phosphate Buffered Saline; KAHRP, Knob-Associated Histidine Rich Protein; GRP(BiP), Glucose-Regulated Protein (Binding protein); BSA, Bovine Serum Albumin; DAPI, 4',6-Diamidino-2-phenylinodole dihydrochloride; MESA, Mature parasite-infected Erythrocyte Surface Antigen; ER, Endoplasmic Reticulum; iRBCs, infected Red Blood Cells; uRBCs, Uninfected Red Blood Cells; KO, Knockout; NPP, New Permeation Pathways; SK, IK and BK, Small, Intermediate and Big Conductance K^+ ^channels, respectively; PEXEL, Plasmodium Export Element; VTS, Vaculolar Transport Signal.

## Authors' contributions

KLW performed the molecular biological analyses, antibody experiments, parasite culture, transfections and analyses, and writing of the manuscript. SMM performed the functional expression experiments. KK was involved in the initial *in silico *analysis, in guidance of the entire project and writing of the manuscript. TVM performed the *in silico *analysis, initial molecular cloning, and directed the project and writing of the manuscript. All authors read and approved the final manuscript.
